# Composition and influencing factors of malnutrition management costs among gastrointestinal cancer patients with chronic comorbidities

**DOI:** 10.3389/fpubh.2026.1868778

**Published:** 2026-08-06

**Authors:** Ronghuan Zhang, Danlu Zhang, Lili Zhao, Yuanyuan Tang, Lixue Cui, Ling Zhou

**Affiliations:** 1 School of Nursing, Ningxia Medical University, Yinchuan, China; 2 Department of Nursing, People's Hospital of Ningxia Hui Autonomous Region, Ningxia Medical University, Yinchuan, China; 3 Department of Radiotherapy, People's Hospital of Ningxia Hui Autonomous Region, Yinchuan, China

**Keywords:** comorbidity, cost of illness, gastrointestinal cancers, health expenditures, malnutrition, nutritional support

## Abstract

**Objective:**

Malnutrition is highly prevalent among patients with gastrointestinal (GI) malignancies and is further aggravated by comorbid chronic conditions, substantially increasing healthcare expenditures. However, few studies have dissected the cost composition of malnutrition management or identified its determinants in this comorbid population. This study aimed to characterize the composition of malnutrition management costs and explore their influencing factors among GI cancer patients with comorbidities.

**Methods:**

A retrospective cohort study was conducted at a tertiary hospital in Yinchuan, China. A total of 418 GI cancer inpatients with at least one chronic comorbidity, diagnosed with malnutrition between October 2024 and October 2025, were enrolled. Data on demographic characteristics, disease profiles, nutritional treatment modalities, and itemized hospitalization costs were extracted from the Hospital Information System (HIS). Malnutrition management costs were categorized into seven domains. Multivariable generalized linear models (Gamma distribution, log link) were fitted to identify independent cost determinants.

**Results:**

The median total malnutrition management cost was USD 833.61 (IQR: 386.21–1,391.72). Pharmaceutical cost constituted the largest share (57.35%), followed by laboratory cost (18.32%). Generalized linear model analysis showed that length of hospital stay was significantly positively associated with total cost: patients hospitalized for 10–19 days and ≥20 days incurred 2.06-fold (β = 0.722, *P* < 0.001) and 3.35-fold (β = 1.210, *P* < 0.001) higher costs, respectively. Surgery (β = 0.263, *P* = 0.005), combined parenteral and enteral nutrition (β = 0.945, *P* < 0.001), and total parenteral nutrition (β = 0.929, *P* < 0.001) were also significantly positively associated with total costs. Comorbidity burden (≥3 chronic diseases: β = 0.247, *P* = 0.003; endocrine and metabolic diseases: β = 0.235, *P* = 0.001) and moderate malnutrition (β = 0.211, *P* = 0.010) showed significant positive associations. Conversely, radiotherapy (β = −0.593, *P* < 0.001) and respiratory comorbidities (β = −0.295, *P* < 0.001) were significantly negatively associated with total costs.

**Conclusion:**

Pharmaceutical cost dominates malnutrition management costs in GI cancer patients with comorbidities. Hospital stay duration, nutritional intervention intensity, and comorbidity burden were independently associated with cost variability. These findings may inform clinicians and health policymakers in efforts to optimize resource allocation.

## 1 Introduction

Gastrointestinal cancers (GI) are a common type of malignant tumor worldwide, including gastric cancer, esophageal cancer, colorectal cancer and pancreatic cancer; their incidence and mortality rates rank among the highest globally (1). This group of patients has a high prevalence of malnutrition, which is severe in nature, and the incidence of multiple co-morbidities is becoming increasingly common (2–4).

Malnutrition can reduce patients' tolerance to treatment, significantly prolong hospital stays and increase healthcare costs (5). The combined effects of co-morbidities and malnutrition further increase the risk of complications, delay recovery and place an additional burden on the healthcare system (6, 7). Nutritional support therapy is a key strategy for improving the nutritional status of patients with gastrointestinal cancer; it can enhance patients' immune function, reduce postoperative complications and increase treatment completion rates (8). Nutritional support therapy involves costs associated with nutritional supplements, pharmacotherapy and nursing procedures, placing a certain financial burden on both patients and the healthcare system. How to optimize the cost composition and control healthcare expenditure whilst ensuring the effectiveness of nutritional support has become a key issue in clinical nutrition management.

Previous studies have largely focused on the clinical efficacy of nutritional support, with little attention paid to the factors influencing its costs; furthermore, they have predominantly concentrated on total hospital costs, and there has been a lack of in-depth analysis of the cost composition of malnutrition management and the factors affecting it. Therefore, investigating the factors influencing the costs associated with the management of malnutrition in patients with co-morbid gastrointestinal cancers, and analyzing the characteristics of the cost composition, provides a basis for optimizing nutritional support strategies, controlling healthcare costs, and alleviating the financial burden on patients.

## 2 Materials and methods

### 2.1 Data source

**Figure 1:**
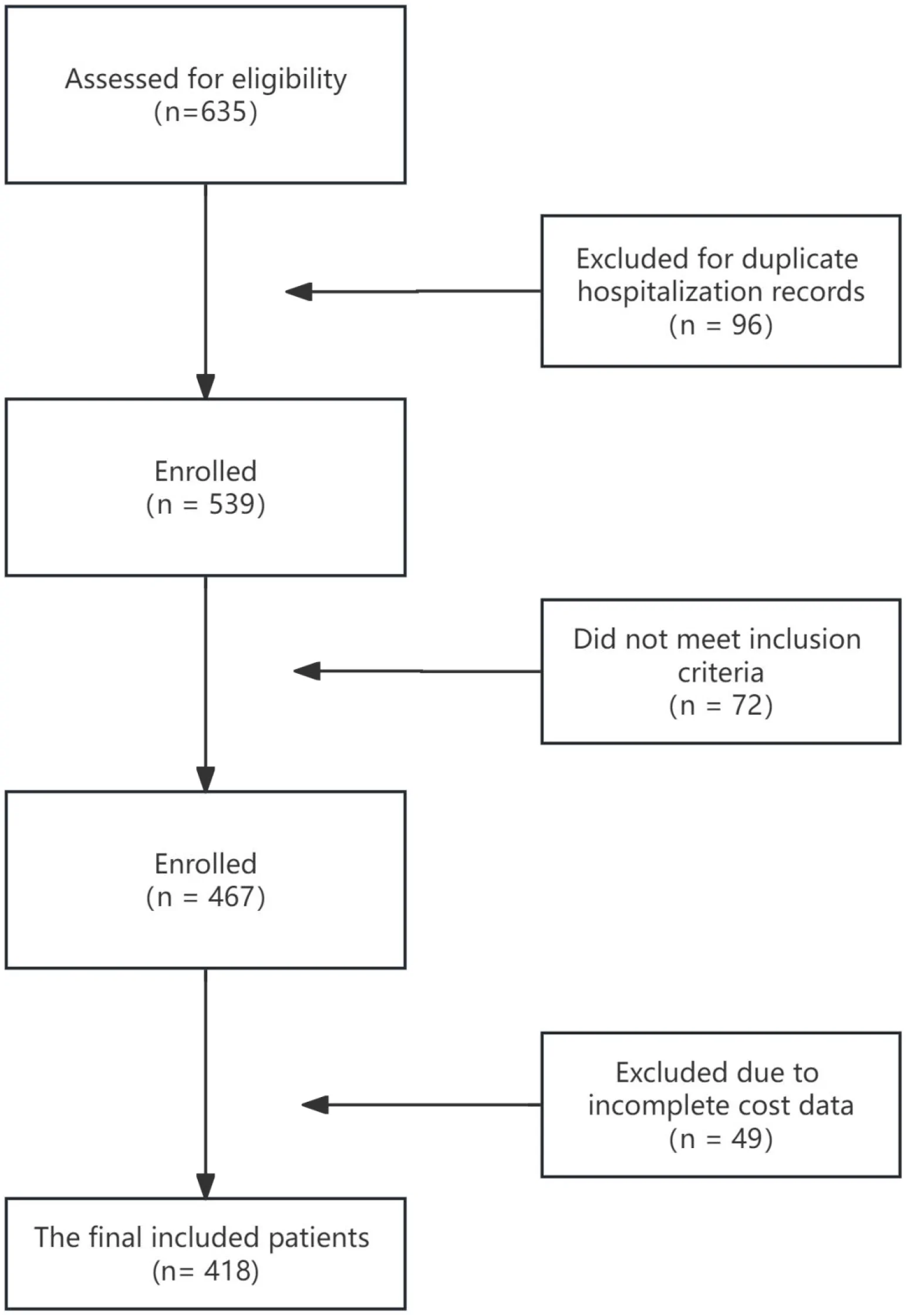
The flow diagram of patient selection (*n* = 418).

This retrospective cohort study enrolled GI cancer patients diagnosed at a tertiary hospital in Yinchuan, China, between October 2024 and October 2025. The inclusion criteria were: (1) pathologically confirmed GI malignancy (esophageal, gastric, duodenal, small intestinal, colorectal, liver, bile duct, gallbladder, or pancreatic cancer); (2) age >18 years; (3) at least one chronic comorbidity in addition to cancer, defined according to the International Classification of Diseases, 10th Revision (ICD-10) (4, 9) documented diagnosis of malnutrition during hospitalization, graded using the Patient-Generated Subjective Global Assessment (PG-SGA); Score: mild = 2~3, moderate = 4~8, severe≥9 (10). Exclusion criteria included: (1) self-discharge before treatment completion; (2) hospital stay < 72 h; and (3) incomplete medical records precluding full cost extraction. A total of 418 patients were enrolled. The patient screening flowchart is shown in Figure 1.

### 2.2 Study instruments

The tool used in this study to estimate the costs of managing malnutrition in patients with co-morbid gastrointestinal cancers was developed based on the following process: (1) Conduct a systematic literature review to establish a standard definition of the costs associated with the management of malnutrition, defining these as the direct medical costs explicitly incurred during hospitalization for the assessment of malnutrition, nutritional support therapy and the management of its complications; and develop a cost classification framework for studies on the costs of nutritional care in cancer patients, both domestically and internationally; (2) Compare the billing items in the HIS system against the hospital's standard operating procedures (SOPs) for nutritional care of patients with gastrointestinal cancer, item by item; (3) Four experts in the fields of clinical nutrition and oncology nursing, holding the title of Associate Senior Physician or above and with over 10 years' professional experience, were invited to review the content to ensure that the checklist covers all categories of nutrition management-related costs encountered in clinical practice.

This study drew upon domestic and international research on the costs associated with malnutrition management in hospitalized patients (5, 11–15) and ultimately defined malnutrition management costs as the direct medical expenses incurred during hospitalization for nutritional assessment, nutritional support therapy, nutritional care, and monitoring and management of nutrition-related complications. Through literature review combined with regional billing policies, the scope of resource utilization related to malnutrition management in cancer patients and the rationale for its cost sources were clarified, and a cost extraction tool was developed based on hospital billing codes. The cost sources comprise two components: (1) Costs related to nutritional therapy: All patients diagnosed with malnutrition undergo a consultation with the Department of Nutrition, where the nutritionist documents a clear nutritional diagnosis and provides a written nutritional treatment plan. The attending physician issues corresponding medical orders based on these consultation findings. This study included only those cost items explicitly recorded as involving nutritional intervention in the medical orders, ensuring that each expense was objectively supported by a nutrition consultation opinion. (2) Costs related to nutritional care: These were identified according to the operational guidelines established by the hospital's specialized nutritional care team. The inclusion criteria required nursing interventions explicitly listed in the guidelines as directly relevant to malnutrition management (e.g., enteral nutrition infusion care, nasogastric tube maintenance care, parenteral nutrition access care), with supporting documentation of care implementation. Cost items solely used for targeted cancer treatments without clear nutritional indications were excluded.

The tool comprises five sections: (1) General information: designed by the researcher, this primarily includes gender, age, BMI, type of health insurance, admission number, length of stay, etc.; (2) Disease characteristics: duration of illness, tumor type, grade and location, chronic conditions, etc.; (3) Nutritional status: malnutrition diagnosis code on the medical record cover sheet; (4) Nutritional therapy modality: records the form of nutritional therapy selected by the patient, such as enteral nutrition or parenteral nutrition; (5) Resources consumed during the management process of malnutrition in patients with co-morbid gastrointestinal cancer (required consumables and medical services), such as costs incurred for the assessment and treatment of malnutrition, including Laboratory cost, medical supplies, Nursing cost, pharmaceutical costs, Treatment cost, Diagnostic examination cost and Other cost.

### 2.3 Data collection

Following approval from the hospital management, and based on the hospital's standard operating procedures for the management of malnutrition in patients with gastrointestinal cancer, the patient's inpatient cost statement was retrieved from the HIS system. Costs associated with malnutrition management were extracted, including only those items explicitly designated for nutritional assessment, monitoring and management. Cost items were extracted and classified according to the predefined criteria described. These included Laboratory cost (e.g., complete blood count, plasma proteins, lipid metabolism, micronutrients, inflammatory markers, urinalysis, stool tests, etc.), Medical consumable cost (e.g. enteral feeding tubes, peripherally inserted central venous catheters, etc.), Nursing cost (e.g. catheter insertion and care fees, etc.), Diagnostic examination cost (e.g., CT scans, gastroscopy, etc.), Treatment cost (e.g., gastrointestinal tube placement, parenteral nutrition therapy, central venous catheterisation, etc.), pharmaceutical costs (e.g., medium- and long-chain triglyceride emulsions, enteral nutrition formulas, multivitamin and mineral supplements, etc.), and Other cost (e.g., consultation fees, personalized nutritional guidance, etc.). The final tally is independently compiled by two researchers who have undergone standardized training, with any discrepancies resolved by a third researcher. To mitigate the risk of overestimation, this study adhered to a conservative inclusion principle: expense items with ambiguous classification were excluded by default in the absence of clear nutritional indications supporting their use.

### 2.4 Statistical analysis

SPSS 27.0 was used for data processing and analysis. Descriptive statistics were applied for demographic characteristics, disease profiles, and cost composition. The Kolmogorov-Smirnov test indicated that total malnutrition management costs did not follow a normal distribution (*D* = 0.134, *P* < 0.001); therefore, median (*M*) and interquartile range (IQR) were used for description. Univariate analysis employed Kruskal-Wallis *H* or Mann-Whitney *U* tests, with Bonferroni correction for multiple comparisons. Multivariable analysis utilized generalized linear models (Gamma distribution with log link function) to identify statistically significant determinants, with *P* < 0.05 considered statistically significant. Variable assignments are presented in Table 1. The variable codes for the influencing factors are as follows: gender (1, male; 2, female); age (1, ≤ 59 years; 2, 60–69 years; 3, 70–79 years; 4, ≥80 years); BMI (1, < 18.5 kg/m^2^; 2, 18.5–23.9 kg/m^2^; 3, ≥24.0 kg/m^2^), Inpatient treatment options (1, supportive care for malignant tumors; 2, chemotherapy; 3, radiotherapy; 4, surgery; 5, Concurrent radiotherapy or chemotherapy during surgery), length of hospital stay (1, < 10 days; 2, 10–19 days; 3, ≥20 days), number of chronic conditions (1, 1 condition; 2, 2 conditions; 3, ≥3 conditions), tumor type (1, upper gastrointestinal tract tumor; 2, lower gastrointestinal tract tumor), Nutritional therapy modality (1, total enteral nutrition; 2, Partial parenteral nutrition combined with enteral nutrition; 3, Total parenteral nutrition), tumor stage (1, Stages I–II; 2, Stage III; 3, Stage IV), malnutrition grade (1, Mild; 2, Moderate; 3, Severe), endocrine and metabolic diseases (0, Absent; 1, Present), respiratory diseases (0, Absent; 1, Present), cardiovascular diseases (0, Absent;1, Present), genitourinary diseases (0, Absent;1, present), non-neoplastic gastrointestinal diseases (0, Absent;1, Present), musculoskeletal diseases (0, Absent; 1, Present).

**Table 1:** Composition of total costs for the management of malnutrition in patients with co-morbid gastrointestinal cancers.

Cost category	*M* (P25, P75)	IQR	Composition, % (95% Cl)
Pharmaceutical cost (USD)	504.08 (188.48, 930.00)	741.52	57.35 (57.29–57.42)
Laboratory cost (USD)	114.22 (69.75, 179.83)	110.08	18.32 (18.28–18.36)
Treatment cost (USD)	55.46 (0.00, 129.02)	129.02	8.45 (8.42–8.48)
Medical consumable cost (USD)	28.70 (16.40, 115.72)	99.32	4.19 (4.18–4.20)
Nursing cost (USD)	37.82 (3.92, 63.59)	59.66	3.92 (3.90–3.93)
Diagnostic examination cost (USD)	28.01 (0.00, 46.92)	46.92	6.95 (6.93–6.97)
Other cost (USD)	0.84 (0.00, 2.87)	2.87	0.77 (0.76–0.77)
total malnutrition management cost (USD)	833.61 (386.21, 1391.72)	1,005.51	/

Amounts are expressed as medians (quartiles); Composition ratios are expressed as means with 95% confidence intervals. All cost data were converted to US dollars based on the 2025 annual average exchange rate (USD 1 = CNY 7.14). Percentages may not sum to exactly 100% due to rounding.

## 3 Results

### 3.1 Composition of malnutrition management costs

The median total malnutrition management cost was USD 833.61 (IQR: 386.21–1,391.72). As shown in Table 1, pharmaceutical cost constituted the predominant cost component (57.35%, 95% CI: 57.29–57.42), with a median of USD 504.08 (IQR: 188.48–930.00), reflecting the substantial expenditure on artificial nutritional formulations required by GI cancer patients with impaired oral intake and hypercatabolic metabolism. Laboratory cost ranked second (18.32%, 95% CI: 18.28–18.36), with a median of USD 114.22 (IQR: 69.75–179.83), indicating intensive nutritional monitoring and biochemical surveillance. Treatment cost (8.45%, 95% CI: 8.42–8.48), diagnostic examination cost (6.95%, 95% CI: 6.93–6.97), medical consumable cost (4.19%, 95% CI: 4.18–4.20), nursing cost (3.92%, 95% CI: 3.90–3.93), and other cost (0.77%, 95% CI: 0.76–0.77) collectively accounted for 22.4%, primarily reflecting the costs of nutritional access establishment, maintenance, and complication management.

### 3.2 Basic characteristics of the participants and univariate analysis of malnutrition management costs

This study enrolled 418 GI cancer patients with comorbidities. The majority were male (70.3%), with a mean age of 66.48 ± 10.64 years, indicating an older adult predominant cohort. The mean BMI was 20.98 ± 3.74 kg/m^2^, reflecting the overall normal-to-underweight nutritional status of this population. Upper GI tumors accounted for 69.38%, and 82.54% of patients presented with stage III–IV disease, suggesting that most patients were diagnosed with malnutrition at intermediate-to-advanced stages. The distribution of malnutrition grade was: mild (31.10%), moderate (30.86%), and severe (38.04%). Combined enteral and parenteral nutrition was the most common nutritional modality (68.90%), and 41.87% of patients underwent surgical treatment. The mean hospital stay was 19.29 ± 11.43 days, and the mean number of chronic comorbidities was 2.12±1.24, indicating substantial comorbidity burden (Table 2).

**Table 2:** General characteristics of patients with co-morbid gastrointestinal cancers, and univariate analysis of total costs, Pharmaceutical cost and Laboratory cost in malnutrition management (*n* = 418).

Variable		Total (%)	Total malnutrition management cost (USD)			Pharmaceutical cost (USD)			Laboratory cost (USD)		
			*M* (P25, P75)	*Z/H*-value	*P*-value	*M* (P25, P75)	*Z/H*-value	*P*-value	*M* (P25, P75)	*Z/H*-value	*P*-value
Gender	Male	294 (70.33)	757.06 (355.63, 1,426.70)	−1.280	0.201	447.67 (183.60, 943.18)	−0.943	0.346	108.12 (69.05, 175.91)	−1.469	0.142
	Female	124 (29.67)	976.04 (479.72, 1,381.81)			631.46 (207.32, 924.83)			126.47 (73.11, 191.88)		
Age (year)	≤ 59	111 (26.56)	946.10 (423.45, 1,427.12)	4.874	0.181	576.22 (210.98, 935.05)	4.368	0.224	114.15 (73.25, 168.91)	1.460	0.694
	60~69	128 (30.62)	847.92 (450.99, 1,367.29)			465.80 (191.42, 918.72)			122.62 (71.22, 189.43)		
	70~79	137 (32.78)	806.76 (383.75, 1,442.95)			519.65 (257.91, 961.03)			115.13 (70.03, 187.54)		
	≥80	42 (10.04)	498.13 (244.87, 1,299.56)			242.93 (111.78, 818.82)			91.39 (58.26, 172.83)		
BMI (kg/m^2^)	< 18.5	122 (29.19)	661.02 (273.95, 1,235.96)	13.495	< 0.001	390.54 (141.50, 914.00)	6.319	0.042	80.11 (53.22, 131.65)	33.936	< 0.001
	18.5~23.9	205 (49.04)	832.61 (404.30, 1,388.93)			524.11 (198.75, 922.86)			119.75 (73.25, 186.13)		
	≥24.	91(21.77)	1,019.41 (634.14, 1,618.09)			615.95 (307.62, 1,064.45)			151.26 (96.92, 209.87)		
Number of chronic conditions	1	109 (26.08)	718.77 (278.85, 1,198.72)	25.33	< 0.001	360.43 (116.10, 870.00)	29.193	< 0.001	92.02 (58.40, 149.30)	17.593	< 0.001
	2	152 (36.36)	721.69 (361.04, 1,175.84)			397.95 (184.25, 708.53)			114.78 (68.77, 164.99)		
	≥3	157 (37.56)	1,171.37 (569.60, 1,660.90)			745.33 (345.56, 1,165.05)			131.65 (79.55, 215.69)		
Length of hospital stay (day)	< 10	59 (14.11)	273.96 (170.65, 450.48)	128.939	< 0.001	146.05 (60.77, 204.21)	92.803	< 0.001	51.54 (38.94, 70.73)	149.059	< 0.001
	10~19	204 (48.80)	752.09 (423.45, 1,166.77)			440.34 (217.43, 782.89)			98.11 (72.27, 145.17)		
	≥20	155 (37.09)	1,388.93 (924.86, 1,997.06)			888.00 (449.70, 1,380.61)			184.45 (125.77,263.87)		
Tumor type	Upper gastrointestinal tract tumor	290 (69.38)	781.24 (346.76, 1,428.02)	−1.365	0.172	483.22 (172.63, 926.79)	−1.596	0.111	103.08 (61.90, 175.35)	−3.120	0.020
	Lower gastrointestinal tract tumor	128 (30.62)	906.22 (560.76, 1,378.43)			604.63 (317.39, 971.27)			132.84 (86.97, 200.63)		
Tumor stage	I~II	73 (17.46)	1,131.84 (669.63, 1,591.19)	19.480	< 0.001	655.85 (323.29, 1,096.19)	10.347	0.006	138.52 (93.70, 210.92)	32.282	< 0.001
	III	152 (36.36)	981.12 (444.35, 1,475.72)			589.24 (197.60, 950.51)			133.96 (78.15, 198.60)		
	IV	193 (46.18)	687.19 (299.39, 1,219.76)			407.60 (135.24, 883.21)			86.13 (58.96, 149.86)		
Malnutrition grade	Mild	130 (31.10)	915.92 (373.15, 1,388.14)	9.016	0.011	558.37 (202.22, 895.69)	7.167	0.280	121.85 (81.51, 168.07)	12.715	0.002
	Moderate	129 (30.86)	1,001.94 (518.43, 1,557.55)			607.68 (198.61, 1,130.64)			129.97 (75.35, 218.07)		
	Severe	159 (38.04)	687.19 (338.88, 1,259.29)			397.95 (159.21, 920.28)			88.24 (57.70, 160.29)		
INPATIENT TREATMENT OPTIONS	Supportive care for malignant tumors	93 (22.25)	551.91 (259.11, 1,044.08)	80.062	< 0.001	345.56 (146.05, 678.54)	58.910	< 0.001	71.71 (51.68, 101.40)	106.029	< 0.001
	Chemotherapy	68 (16.27)	613.29 (341.62, 1,213.93)			377.93 (133.08, 852.40)			86.55 (55.18, 137.82)		
	Radiotherapy	31 (7.41)	303.63 (201.65, 653.68)			109.31 (29.37, 347.41)			65.97 (55.81, 80.11)		
	Surgery	175 (41.87)	1,219.76 (799.55, 1,617.66)			726.22 (410.19, 1,094.35)			156.58 (112.96,228.36)		
	Surgery concurrent radiotherapy or chemotherapy during surgery	51 (12.20)	827.43 (417.15, 1,339.29)			437.76 (236.54, 855.38)			122.13 (88.80, 186.90)		
Nutritional therapy modality	total enteral nutrition	28 (6.70)	199.49 (94.50, 345.08)	48.856	< 0.001	37.99 (12.94, 64.74)	64.335	< 0.001	62.18 (39.43, 87.82)	17.106	< 0.001
	Partial parenteral nutrition combined with enteral nutrition	288 (68.90)	925.66 (476.48, 1,485.70)			577.37 (250.88, 974.59)			122.97 (74.30, 189.64)		
	Total parenteral nutrition	102 (24.40)	859.25 (525.47, 1,312.21)			506.17 (230.96, 932.94)			108.82 (72.83, 172.83)		
Endocrine and metabolic diseases	Absent	302 (72.25)	738.44 (334.39, 1,273.27)	−4.952	< 0.001	408.20 (152.92, 855.18)	−5.402	< 0.001	107.42 (60.92, 162.89)	−4.237	< 0.001
	Present	116 (27.75)	1,171.37 (630.93, 1,743.03)			734.50 (391.40, 1,289.89)			146.22 (86.06, 238.87)		
Respiratory diseases	Absent	359 (85.89)	862.05 (399.82, 1,427.12)	−2.146	0.032	384.03 (161.67, 798.93)	−1.806	0.710	114.29 (71.43, 183.47)	−1.106	0.309
	Present	59 (14.11)	607.27 (297.66, 1,253.92)			522.80 (195.27, 942.57)			112.89 (58.61, 157.21)		
Cardiovascular diseases	Absent	237 (57.70)	880.86 (417.41, 1,388.14)	−0.163	0.871	480.33 (197.69, 926.79)	−0.180	0.857	112.89 (71.57, 166.67)	−0.841	0.400
	Present	181 (43.30)	800.29 (377.35, 1,400.11)			522.07 (183.60, 931.88)			116.39 (69.05, 197.90)		
Genitourinary diseases	Absent	383 (91.63)	834.61 (389.52, 1,413.41)	−0.056	0.955	505.64 (186.10, 932.41)	−0.116	0.907	114.15 (70.45, 175.77)	−0.541	0.589
	Present	35 (8.37)	789.58 (406.62, 1,367.68)			359.29 (197.91, 882.76)			136.97 (62.96, 216.32)		
Gastrointestinal diseases	Absent	166 (39.71)	799.55 (324.91, 1,445.87)	−0.689	0.491	478.10 (158.42, 954.40)	−0.236	0.814	114.15 (68.21, 192.58)	−0.023	0.982
	Present	252 (60.28)	856.03 (436.34, 1,385.99)			513.81 (198.68, 928.11)			114.22 (71.08, 176.82)		
Musculoskeletal diseases	Absent	396 (94.74)	841.56 (393.86, 1,428.10)	−1.440	0.150	408.26 (161.94, 755.84)	−1.102	0.270	115.90 (71.64, 185.29)	−2.328	0.020
	Present	22 (5.26)	748.72 (300.15, 984.77)			513.17 (191.42, 942.57)			97.13 (43.00, 130.95)		

^*^For binary variables, the Mann-Whitney U test (two-sample rank-sum test) was used, with the *Z*-statistic, to compare differences in the distribution of nutritional costs between two groups of patients; for multi-category variables, the Kruskal-Wallis *H* test (multi-sample rank-sum test) was used, with the H-statistic, to compare differences in the distribution of nutritional costs among three or more groups of patients. A *p*-value of < 0.05 was considered statistically significant. All cost data were converted to US dollars based on the 2025 annual average exchange rate (USD 1 = CNY 7.14).

Univariate analysis (Table 2) revealed that Length of hospital stay, nutritional therapy modality, treatment modality, number of chronic diseases, endocrine and metabolic diseases, respiratory diseases, tumor stage, BMI, and malnutrition grade were significantly associated with total malnutrition management cost (*P* < 0.05). For pharmaceutical costs, significant associations were observed with Length of hospital stay, nutritional therapy modality, treatment modality, number of chronic diseases, endocrine and metabolic diseases, and tumor stage (*P* < 0.05). For Laboratory costs, significant associations were identified with Length of hospital stay, nutritional therapy modality, treatment modality, number of chronic diseases, endocrine and metabolic diseases, musculoskeletal diseases, tumor stage, BMI, malnutrition grade, and tumor type (*P* < 0.05). Notably, Length of hospital stay exhibited the strongest effect across all three cost outcomes (H = 128.939 for total cost, *P* < 0.001).

### 3.3 Multivariable generalized linear model analysis

Generalized linear models (GLMs) are effective for analyzing data that do not follow a normal distribution (16); therefore, generalized linear models (with a Gamma distribution and a logarithmic link function) were employed for the multivariate analysis.To identify independent cost determinants while mitigating overfitting, a two-step variable selection strategy was adopted: variables with *P* < 0.10 in univariate analysis and those deemed clinically important were retained as candidate predictors, followed by manual backward stepwise selection based on Akaike Information Criterion (AIC). The final total-cost model included 14 predictors (AIC = 7,963.48), the pharmaceutical cost model retained 13 predictors (AIC = 7,687.22), and the laboratory cost model included 15 predictors (AIC = 6,224.24). All models showed good fit (Omnibus *P* < 0.001; Pearson χ^2^/df < 1) and no multicollinearity (VIF < 2).

For total malnutrition management cost (Table 3), the model revealed that length of hospital stay was the strongest independent determinant: patients hospitalized for 10–19 days incurred 2.06-fold higher costs (β = 0.722, 95% CI: 0.523–0.920, *P* < 0.001), while those hospitalized for ≥20 days incurred 3.35-fold higher costs (β = 1.210, 95% CI: 1.000–1.420, *P* < 0.001), compared to the reference group (< 10 days). Surgery was also significantly positively associated with total cost (β = 0.263, 95% CI: 0.077–0.449, *P* = 0.005), corresponding to an approximately 30.1% increase. Nutritional therapy modality showed substantial effects: combined enteral and parenteral nutrition (β = 0.945, 95% CI: 0.691–1.200, *P* < 0.001) and total parenteral nutrition (β = 0.929, 95% CI: 0.672–1.185, *P* < 0.001) were associated with 2.57-fold and 2.53-fold higher costs, respectively, relative to exclusive enteral nutrition.

**Table 3:** Generalized linear model estimates for total, pharmaceutical, and laboratory costs of malnutrition management (*n* = 418).

Dependent variable	Independent variable		β	SE	Wald χ^2^-value	*P*-value	95% CI
Total malnutrition management cost	Intercept		6.785	0.173	1,546.864	< 0.001	6.447~7.123
	Length of hospital stay (ref: < 10 days)	10~19 days	0.722	0.101	50.819	< 0.001	0.523~0.920
		≥20 days	1.210	0.107	127.684	< 0.001	1.000~1.420
	Nutritional therapy modality (ref: total enteral nutrition)	Partial parenteral nutrition combined with enteral nutrition	0.945	0.130	52.868	< 0.001	0.691~1.200
		Total parenteral nutrition	0.929	0.131	50.407	< 0.001	0.672~1.185
	Inpatient treatment options (ref: supportive care for malignant tumors)	Chemotherapy	−0.002	0.118	0.000	0.99	−0.232~0.229
		Radiotherapy	−0.593	0.146	16.48	< 0.001	−0.879~-0.307
		Surgery	0.263	0.095	7.715	0.005	0.077~0.449
		Surgery Concurrent radiotherapy or chemotherapy during surgery	0.069	0.108	0.410	0.522	−0.142~0.281
	Number of chronic conditions (ref: 1 condition)	2 conditions	−0.032	0.075	0.180	0.671	−0.180~0.116
		≥3 conditions	0.247	0.084	8.564	0.003	0.081~0.412
	Endocrine and metabolic diseases (ref: absent)	Present	0.235	0.072	10.642	0.001	0.094~0.377
	Respiratory diseases (ref: absent)	Present	−0.295	0.078	14.217	< 0.001	−0.448~-0.141
	Malnutrition grade (ref: mild)	Moderate	0.211	0.082	6.586	0.010	0.050~0.372
		Severe	0.081	0.071	1.315	0.251	−0.057~0.219
Pharmaceutical cost	Intercept		5.412	0.208	678.636	< 0.001	5.004~5.819
	Length of hospital stay (ref: < 10 days)	10~19 days	0.774	0.124	38.783	< 0.001	0.530~1.018
		≥20 days	1.257	0.137	84.815	< 0.001	0.990~1.525
	Nutritional therapy modality (ref: total enteral nutrition)	Partial parenteral nutrition combined with enteral nutrition	1.961	0.170	133.632	< 0.001	1.629~2.294
		Total parenteral nutrition	1.955	0.176	124.107	< 0.001	1.611~2.299
	Inpatient treatment (ref: supportive care for malignant tumors)	Chemotherapy	0.084	0.129	0.428	0.513	−0.169~0.337
		Radiotherapy	−0.801	0.174	21.1	< 0.001	−1.142~-0.460
		Surgery	0.173	0.112	2.396	0.122	−0.047~0.393
		Surgery Concurrent radiotherapy or chemotherapy during surgery	−0.059	0.143	0.172	0.678	−0.339~0.221
	Number of chronic conditions (ref: 1 condition)	2 conditions	−0.132	0.099	1.770	0.183	−0.326~0.062
		≥3 conditions	0.259	0.100	6.668	0.010	0.063~0.455
	Endocrine and metabolic diseases (ref: absent)	Present	0.311	0.091	11.56	0.001	0.133~0.489
	tumor stage (ref: stages I–II)	Stage III	−0.022	0.117	0.036	0.850	−0.251~0.207
		Stage IV	0.001	0.114	0.000	0.992	−0.222~0.224
Laboratory cost	Intercept		5.567	0.088	3,986.627	< 0.001	5.395~5.740
	Length of hospital stay (ref: < 10 days)	10~19 days	0.537	0.068	62.403	< 0.001	0.404~0.671
		≥20 days	1.009	0.077	172.971	< 0.001	0.858~1.159
	Inpatient treatment options (ref: supportive care for malignant tumors)	chemotherapy	0.116	0.095	1.489	0.222	−0.071~0.303
		radiotherapy	−0.259	0.109	5.663	0.017	−0.473~-0.046
		surgery	0.409	0.069	35.756	< 0.001	0.275~0.548
		Surgery Concurrent radiotherapy or chemotherapy during surgery	0.225	0.079	8.165	0.004	0.071~0.379
	Number of chronic conditions (ref: 1 condition)	2 conditions	0.036	0.058	0.376	0.540	−0.078~0.149
		≥3 conditions	0.210	0.069	9.329	0.002	0.075~0.345
	Endocrine and metabolic diseases (ref: absent)	Present	0.199	0.059	11.379	0.001	0.084~0.315
	Musculoskeletal diseases (ref: absent)	Present	−0.318	0.074	18.263	< 0.001	−0.463~-0.172
	Malnutrition grade (ref: mild)	Moderate	0.227	0.063	13.149	< 0.001	0.104~0.349
		Severe	0.111	0.056	4.013	0.045	0.002~0.220
	BMI group (ref: < 18.5 kg/m^2^)	18.5~23.9 kg/m^2^	0.113	0.056	4.110	0.043	0.004~0.223
		≥24.0 kg/m^2^	0.208	0.066	9.936	0.002	0.079~0.337
	Tumor type (ref: upper GI tract tumor)	Lower GI tract tumor	0.145	0.057	6.506	0.011	0.033~0.256

^*^All models used Gamma distribution with log link and maximum likelihood scale estimation. Total cost: df = 14, AIC = 7,963.477, Pearson χ^2^/df = 0.363, Deviance/df = 0.364. Pharmaceutical cost: df = 13, AIC = 7,687.222, Pearson χ^2^/df = 0.646, Deviance/df = 0.674. Laboratory cost: df = 15, AIC = 6,224.242, Pearson χ^2^/df = 0.265, Deviance/df = 0.256. All Omnibus tests *P* < 0.001. VIF, Scale parameters: 0.333, 0.646, 0.237. The VIF values for all models are < 2. a. Parameter set to zero due to redundancy. B, Maximum likelihood estimate.

Comorbidity burden emerged as a significant cost driver. Patients with ≥3 chronic diseases had 1.28-fold higher total costs (β = 0.247, 95% CI: 0.081–0.412, *P* = 0.003) compared to those with a single comorbidity, and patients with endocrine and metabolic diseases incurred 1.27-fold higher costs (β = 0.235, 95% CI: 0.094–0.377, *P* = 0.001). Conversely, radiotherapy was significantly negatively associated with total cost (β = −0.593, 95% CI: −0.879 to −0.307, *P* < 0.001), with costs approximately 55.3% of those in the supportive care reference group. Respiratory comorbidities were also significantly negatively associated (β= −0.295, 95% CI: −0.448 to −0.141, *P* < 0.001), corresponding to approximately 74.4% of reference costs. Chemotherapy (β = −0.002, *P* = 0.990) and surgery concurrent with chemoradiotherapy β = 0.069, *P* = 0.522 were not significantly associated with total costs.

For pharmaceutical costs (Table 3), the model identified length of hospital stay, combined enteral and parenteral nutrition β = 1.961, *P* < 0.001, total parenteral nutrition β = 1.955, *P* < 0.001, ≥3 chronic diseases β = 0.259, *P* = 0.010, and endocrine and metabolic diseases β = 0.311, *P* = 0.001 as significant positive determinants. The effect sizes for nutritional therapy modality were particularly pronounced: combined and total parenteral nutrition were associated with approximately 7.11-fold and 7.06-fold higher pharmaceutical costs, respectively, compared to exclusive enteral nutrition. Radiotherapy was significantly negatively associated with pharmaceutical cost (β = −0.801, 95% CI: −1.142 to −0.460, *P* < 0.001). Tumor stage did not retain significance in this model (Stage III: β = −0.022, *P* = 0.850; Stage IV: β = 0.001, *P* = 0.992).

For laboratory costs (Table 3), length of hospital stay, surgery β = 0.409, *P* < 0.001, surgery combined with chemoradiotherapy β = 0.225, *P* = 0.004, ≥3 chronic diseases β = 0.210, *P* = 0.002, endocrine and metabolic diseases β = 0.199, *P* = 0.001, moderate malnutrition β = 0.227, *P* < 0.001, severe malnutrition β = 0.111, *P* = 0.045, BMI 18.5–23.9 kg/m^2^ β = 0.113, *P* = 0.043, BMI ≥24.0 kg/m^2^ β = 0.208, *P* = 0.002, and lower GI tumor β = 0.145, *P* = 0.011were all significantly positively associated. Musculoskeletal comorbidities were significantly negatively associated (β = −0.318, 95% CI: −0.463 to −0.172, *P* < 0.001). Notably, nutritional therapy modality did not retain significance in this model and was excluded in the AIC-optimized model, suggesting that laboratory monitoring intensity was independent of the nutritional access route.

## 4 Discussion

This study reveals that Pharmaceutical cost constitutes the predominant cost driver, accounting for nearly two-thirds (64.0%) of total malnutrition management costs. This finding aligns with the pathophysiological characteristics of GI malignancies: tumor-related mechanical obstruction, impaired digestion and absorption, systemic inflammatory response, and metabolic dysregulation collectively limit oral intake and sustain a hypercatabolic state, necessitating substantial reliance on artificial nutritional formulations (17). Laboratory cost ranked second (13.6%), reflecting intensive nutritional monitoring, consistent with the findings of multiple scholars (18, 19). This is consistent with ESPEN guidelines, which recommend regular assessment of nutritional intake, weight changes, BMI, muscle mass, and systemic inflammation in cancer patients (3), and with the widespread application of prognostic nutritional index (PNI) and CONUT scores in GI cancer nutritional evaluation (4). While these monitoring activities increase expenditures, they provide essential clinical value by enabling timely identification of malnutrition grade and guiding subsequent nutritional interventions. The relatively lower proportions of therapeutic fees, Medical consumable cost, nursing care, and diagnostic imaging collectively reflect the infrastructure costs of nutritional access establishment and maintenance rather than the primary drivers of expenditure. Treatment and medical supplies costs cover the catheterisation procedure and catheter materials; variations in cost stem from the choice of nutritional access route. Nursing costs are related to the nutritional access route and the severity of the patient's condition; standardized catheterisation care can effectively reduce the risk of catheter-associated infections and unplanned catheter removal. Diagnostic costs primarily comprise imaging examinations to confirm catheter placement or diagnose complications.

Length of hospital stay emerged as a consistently significant independent determinant across all three cost models, consistent with findings from Niu et al. (5). Length of hospital stay reflects greater disease complexity and more severe metabolic disturbances, which typically necessitate higher-intensity nutritional interventions (20). The duration of hospital stay directly determines the cumulative duration of nutritional support, with increasing consumption of nutritional formulations, periodic biochemical monitoring, and elevated risks of nutrition-related complications (21). These findings suggest that early nutritional rehabilitation and streamlined discharge planning may be associated with reduced costs, without compromising patient safety or treatment efficacy (22).

Nutritional therapy modality was associated with cost effects, particularly on pharmaceutical expenditure. Both partial parenteral nutrition combined with enteral nutrition and total parenteral nutrition were independently associated with higher total malnutrition management costs and pharmaceutical costs compared with exclusive enteral nutrition, consistent with prior findings (23). This reflects the substantially greater expenditure on parenteral formulations, catheter materials, and complication management. Parenteral nutrition requires central or peripheral venous access establishment and maintenance, with ongoing costs for catheter insertion, care, dressing changes, and management of catheter-related infections; combined nutrition necessitates simultaneous management of both enteral and parenteral access routes, generating additional resource consumption. However, this association should be interpreted with caution given the potential for confounding by indication. In clinical practice, nutritional route selection is determined by the treating physician based on the patient's gastrointestinal function and overall disease severity (24). Patients with enteral feeding intolerance or greater illness severity are more likely to receive parenteral or combined nutritional support, whereas those with normal gastrointestinal function are generally prioritized for enteral nutrition. Consequently, patients receiving parenteral or combined nutritional support often present with higher disease severity, more complex clinical conditions, and greater complication risk (25, 26), factors that independently drive increased healthcare resource utilization. Previous studies have demonstrated that enteral nutrition reduces infectious complications and shortens hospital length of stay when gastrointestinal function is preserved (27), yet these benefits are contingent upon appropriate patient selection and tolerance.

Comorbidity burden, specifically the presence of ≥3 chronic diseases and endocrine/metabolic disorders, was a significant positive determinant across all three cost models. Multimorbidity increases nutritional management complexity through additional monitoring requirements, altered treatment tolerance, and potential modifications to standard antineoplastic protocols, which may prolong hospitalization and increase nutritional formulation consumption (6, 28). The increased usage of nutritional formulations has led to a corresponding rise in the cost of Pharmaceutical cost and total healthcare expenditures, further exacerbating the burden on the medical system (7). Diabetes mellitus and other endocrine/metabolic disorders directly influence glucose and lipid metabolism and protein synthesis, necessitating more refined nutritional management including glycemic monitoring, insulin adjustment, and specialized enteral formulations (29, 30), all of which contribute to cost escalation.

A striking finding of this study is the relationship between malnutrition grade and management costs. Moderate malnutrition was significantly positively associated with total and Laboratory costs, while severe malnutrition was not significantly associated with total costs in the multivariable model, but remained positively associated with laboratory costs. This divergence may be attributable to the limited capacity for nutritional intervention escalation in severely malnourished patients.

According to the five-step treatment approach for malnutrition (31), mildly malnourished patients typically receive oral nutritional supplements at relatively low cost. Moderately malnourished patients, however, may require escalation from oral nutritional supplements to enteral nutrition, or from enteral nutrition alone to combined partial parenteral and enteral nutrition, as their condition progresses. This escalation process is accompanied by the accumulation of treatment-related expenses—including nasogastric tube placement, central venous catheterization, and associated nursing and consumable costs—which collectively drive total costs upward. Additionally, moderately malnourished patients are in a dynamic phase of nutritional status, necessitating baseline assessment and efficacy monitoring at each intervention escalation step. The resultant increase in testing frequency contributes to the rise in laboratory costs.

In contrast, severely malnourished patients frequently present with gastrointestinal dysfunction, encompassing impaired intestinal motility, mucosal atrophy, and diminished absorptive capacity (32, 33). Nutritional interventions may be unable to advance or escalate due to patient intolerance or underlying gastrointestinal dysfunction. These constraints limit the marginal growth of treatment-related procedural, consumable, and nursing costs, thereby suppressing further accumulation of total malnutrition management costs. On the other hand, severely malnourished patients carry a substantially elevated risk of refeeding syndrome (34), and frequently present with electrolyte disturbances such as hypophosphatemia, hypokalemia, and hypomagnesemia. These acute electrolyte imbalances can precipitate severe complications with adverse effects on patient recovery (35), necessitating sustained and intensified laboratory monitoring until metabolic stability is achieved; consequently, laboratory costs continue to rise.

This study also found that Tumor type significantly influences laboratory testing costs, which may be related to the need for close evaluation of intestinal functional recovery and enteral nutrition tolerance in patients with lower gastrointestinal tumors during treatment (36, 37). Increased monitoring frequency consequently leads to higher testing expenses. BMI categories were positively associated with laboratory costs. This likely reflects the increasing diagnostic complexity of malnutrition assessment at higher BMI levels and the inherent limitations of BMI as a nutritional screening tool (38). In underweight patients, malnutrition can be initially identified through physical examination, with relatively less reliance on biochemical validation. However, when BMI falls within the normal or overweight range, nutritional status cannot be distinguished by simple anthropometric measures, given that BMI does not capture body composition, muscle mass, or visceral protein reserves (39). Consequently, clinicians must rely on laboratory biomarkers to confirm nutritional status. These findings suggest that a single anthropometric measure is insufficient to comprehensively assess nutritional status in patients with gastrointestinal cancer, supporting the use of multidimensional nutritional assessment tools that integrate anthropometry, biochemical markers, and clinical evaluation in practice.

Surgery was significantly positively associated with total and Laboratory costs. Perioperative patients require intensive nutritional support and dense biochemical monitoring of albumin, prealbumin, electrolytes, and hepatic/renal function to assess surgical tolerance and recovery status (40, 41). The initiation of perioperative nutritional intervention has been demonstrated to improve prognostic outcomes (42, 43), findings that contextualize the associated cost increases within the framework of clinically necessary investments. Conversely, radiotherapy was significantly negatively associated with total and pharmaceutical costs, likely because radiotherapy patients predominantly receive outpatient-based care with shorter hospitalization and less intensive nutritional support. International nutritional guidelines consistently recommend oral nutritional supplementation as the first-line intervention for radiotherapy patients (44, 45), which inherently limits nutritional therapy costs. Furthermore, the observed association may be influenced by selection bias and disease stage distribution. Patients receiving radiotherapy typically have earlier disease stages or fewer comorbidities, both of which independently reduce nutritional requirements and overall treatment costs.

Respiratory comorbidities were significantly negatively associated with total costs, While this finding may partly reflect restrictive feeding strategies commonly employed to maintain an appropriate respiratory quotient in patients with compromised respiratory function, patients with compromised respiratory function often require low-volume, high-energy density formulations or restricted total energy intake to maintain an appropriate respiratory quotient (46), resulting in lower standard nutritional intervention intensity. However, reduced nutritional intervention in patients with respiratory diseases may increase the risk of malnutrition, which is closely associated with impaired immune status, elevated infection risks, and prolonged hospitalization for respiratory conditions. Thus, the observed lower costs may reflect potential adverse consequences of inadequate treatment rather than representing the optimal clinical therapeutic approach. Musculoskeletal comorbidities were negatively associated with Laboratory costs (*P* < 0.001, β = −0.318, 95% CI: −0.463 to −0.172), possibly because nutritional status assessment in these patients relies more on physical measurements than intensive biochemical monitoring. This result should be interpreted with caution given the limited sample size (*n* = 22, 5.26%), as the limited sample size may compromise the stability and generalizability of the estimate.

This study identified several factors associated with lower malnutrition management costs; however, the cost reduction cannot be unequivocally attributed to improved medical efficiency or insufficient intensity of nutritional interventions. When nutritional support is restricted or omitted, patients may face nutritional risks such as progressive weight loss, impaired immune function, and reduced treatment adherence, which could subsequently lead to higher healthcare expenditures. Future research should incorporate patient-centered outcome measures—including dynamic changes in nutritional status, treatment completion rates, complication incidence, quality of life, and readmission rates into the analytical framework to avoid cost-centric interpretations.

Gastrointestinal cancers account for a significant proportion of the global cancer burden, and malnutrition and comorbidities are prevalent in this population. The combined effect of malnutrition and comorbidities further exacerbates the strain on healthcare resources. Cohort characteristics of this studys share similarities with national epidemiological data from China. A study by LU et al. (47) reported that the crude incidence rate among male patients was approximately 2.16 times higher than that of female patients. Additionally, a survey on nutritional status among patients with common malignant tumors in China revealed that 58.2% of hospitalized cancer patients suffered from moderate to severe malnutrition (2), particularly among those with gastrointestinal tumors. The predominant comorbidities were endocrine and metabolic disorders, highlighting a substantial comorbidity burden, which aligns with the findings of Wang et al. (48). From a public health perspective, these findings offer preliminary evidence that may inform healthcare decision-making. Multi-center external validation will be required in the future.

This study has several limitations. First, as a single-center retrospective study at a tertiary hospital in northwestern China, regional variations may limit the direct generalizability of our cost estimates; second, only inpatient costs were captured, as Inciong et al. (51) noted, estimates based solely on inpatient costs represent a conservative estimate of the overall burden. Third, long-term outcomes, quality of life, and survival data were not assessed, precluding cost-effectiveness evaluation. Finally, the musculoskeletal comorbidities subgroup was small (*n* = 22, 5.26%), requiring cautious interpretation. Future multicenter prospective studies incorporating linked inpatient-outpatient cost data are warranted.

## 5 Conclusion

This study conducted a retrospective analysis of 418 inpatients with gastrointestinal cancer and comorbidities, revealing the cost composition and key influencing factors of malnutrition management in a real-world clinical setting. The study indicates that expenditure on medication is the primary cost driver, accounting for approximately two-thirds of the total cost of malnutrition management. This suggests that optimisation of the nutritional product formulary and standardized strategies for nutritional support warrant consideration, with attention to reducing potentially unnecessary parenteral nutritional product use. Length of hospitalization and intensity of nutritional interventions were significantly associated with cumulative costs, suggesting that early initiation of nutritional rehabilitation therapy and prioritization of enteral nutrition regimens for eligible patients can help control healthcare expenditures. Local regulatory authorities are advised to tailor malnutrition intervention strategies based on regional characteristics, nutritional treatment needs, and clinical capabilities across different healthcare institutions.

## Data Availability

The original contributions presented in the study are included in the article/supplementary material, further inquiries can be directed to the corresponding author.
